# Intravascular large B-cell lymphoma of the kidney: A case report

**DOI:** 10.1186/1746-1596-6-86

**Published:** 2011-09-23

**Authors:** Xiaoyan Bai, Xiao Li, Liyan Wan, Guobao Wang, Nan Jia, Jian Geng

**Affiliations:** 1 Guangdong Provincial Institute of Nephrology, Nanfang Hospital, Southern Medical University, Guangzhou, Guangdong, 510515, P. R. China; 2 Department of Emergency, Nanfang Hospital, Southern Medical University, Guangzhou, Guangdong, 510515, P. R. China; 3 Department of Pathology, Nanfang Hospital, Southern Medical University, Guangzhou, Guangdong, 510515, P. R. China

**Keywords:** Kidney, Intravascular large B-cell lymphoma, Renal biopsy

## Abstract

We report a 41-year-old Chinese woman with intravascular large B-cell lymphoma diagnosed by percutaneous renal biopsy. The patient was admitted to Nanfang Hospital of Southern Medical University, Guangzhou, China with complaints of high spiking fever for a month and bilateral lower limb fatigue with difficulty ambulating for the past 5 months.

She had renal dysfunction with a total urinary protein of 5.61 g/dL (56.1 g/L), serum albumin of 2.89 g/dL (28.9 g/L), urea nitrogen of 2.24 mg/dL (1.6 mmol/L), and serum creatinine of 0.54 mg/dL (48 μmol/L). Bone marrow biopsy revealed myeloproliferative disorder without abnormal myeloid or lymphocytic proliferation. Positron Emission Tomography-Computed Tomography (PET-CT) showed marked bilateral swelling and enlargement of the renal parenchyma with splenic enlargement and involvement of multiple vertebrae. Percutaneous renal biopsy showed island-like accumulations of medium to large lymphoid cells in many areas of the interstitium, with round vesicular nuclei containing distinct basophilic nucleoli. Immunohistochemical analysis together with other supportive investigation confirmed the diagnosis of intravascular large B-cell lymphoma. Ten days later, she was started on chemotherapy with CHOP (cyclophosphamide, doxorubicin, leurocristime and prednisone) for a week. Palliative radiotherapy DT 40Gy/20F with other supportive treatment was provided for metastatic foci in the medullary cavity of the sternum, T1-T7. The patient regained muscle strength in both lower limbs and was able to walk again after three weeks. The patient was discharged after hepatic and renal function and proteinuria values had returned to normal. Follow-up data shows the patient to be alive nine months after discharge.

## Background

Intravascular large B-cell lymphoma (IVLBCL) is a rare variant of large B-cell lymphoma characterized by proliferation of malignant lymphoid cells in small vessels of organs such as skin, kidneys, adrenals, lungs, liver and the central nervous system. Neurologic findings and skin manifestations may present a clue clinically, while fever of unknown origin without weight loss is a common non-specific symptom. Since Jothy et al first reported a case of IVLBCL in kidney in 1981 [1], seventeen additional cases have been published in the literature [2,3]. Most of these cases had malignant lymphoid cells localized within the glomerular capillaries. One case reported the localization of lymphoma cells within peritubular capillaries [4].

We report here a case with markedly enlarged kidney showing clustering of lymphoma cells within the peritubular capillaries.

## Case presentation

A 41-year-old Chinese woman was referred to the Department of Neurology, Nanfang Hospital of Southern Medical University, Guangzhou, China, with the chief complaint of high spiking fever for approximately a month with bilateral lower limb fatigue and difficulty in defecation for more than five months. She had been residing in Yingde City of Guangdong Province for 10 years with no known prior exposure to any infectious or occupational diseases. In July 2010, she was diagnosed with 'whole body multiple metastases of lymphoma'. Due to economic reasons, the patient was transferred at the end of July 2010 to Donghua Hospital of Yingde City for treatment of her symptoms of infection, constipation, low potassium and leukopenia. By August 2010, the patient was unable to walk with bilateral lower limb fatigue and was subsequently admitted to the Department of Radiation Oncology at Nanfang Hospital.

On admission, the patient was 152 cm tall and weighed 52 kg. Her blood pressure was 112/82 mm Hg, temperature 38.5°C with a respiratory rate of 16 times per minute. No superficial enlarged lymph nodes were palpable. Inspiratory and expiratory sounds were normal to auscultation. There was no percussion tenderness over the liver or kidney. Muscle strength of upper limbs was normal and that of lower limbs was 3/5. Bilateral Babinski sign was positive. Sensation of pain and touch below the knees were slightly reduced. Physiological reflexes were slightly active. There was no pedal edema. All other physical tests were normal.

Laboratory findings were as follows: total urinary protein, 5.61 g/dL (56.1 g/L); serum albumin, 2.89 g/dL (28.9 g/L); blood urea nitrogen, 2.24 mg/dL (1.6 mmol/L); and creatinine, 0.54 mg/dL (48 μmol/L). The urine protein and occult blood values were negative. Urinary leukocyte count was 48/μL (4.8 × 10^7^/L); erythrocyte count, 3.78 × 10^6^/μL (3.78T/L); hemoglobin, 9.9 g/dL (99 g/L); lymphocyte count, 1.12 × 10^3^/μL (1.12 g/l) and platelet count, 1.73 × 10^5^/μL (173 g/l).

**Figure 1:**
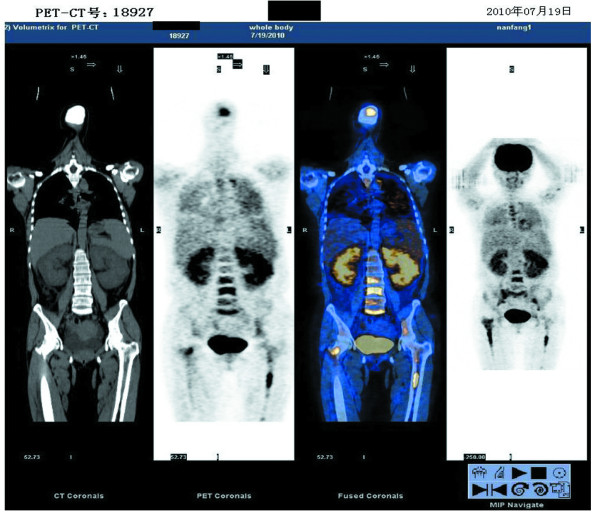
**PET-CT shows foci with high signal intensity in bilateral kidneys, multiple vertebra, bilateral sacrum and ilium**.

Bone marrow aspiration was performed and the slide smear revealed 41.5% immature cells of unknown origin. Positron Emission Tomography-Computed Tomography (PET-CT) showed the following changes (Figure 1). The bilateral renal parenchyma was markedly swollen with obvious diffuse increase in signal intensity. Increased signal intensity was observed in multiple thoracic vertebral bodies, multiple lumbar vertebral bodies, bilateral sacrum, ilium, proximal humerus and femur medullary cavities. Mildly increased signal intensity was observed in both lungs. The possibility of interstitial pneumonia was not excluded. Multiple bilateral neck and mediastinum lymph nodes were mildly enlarged with inflammatory proliferation. The spleen was markedly enlarged with mild diffuse increase in signal intensity. A large volume of pelvic effusion was detected.

**Figure 2:**
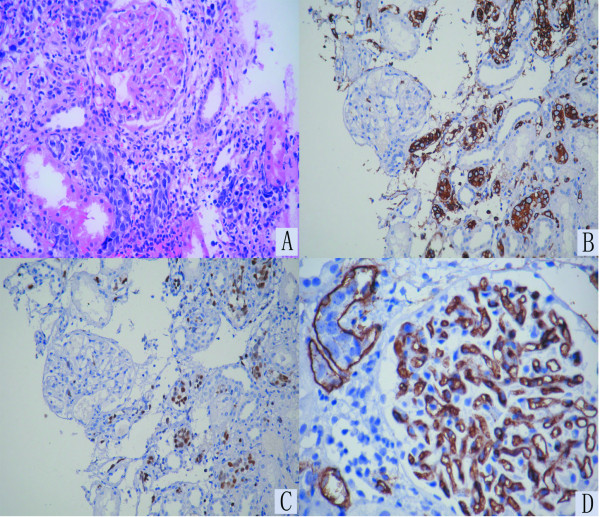
**Hematoxylin & Eosin (H&E) shows mild changes in glomeruli**. Island-like infiltrations of medium to large atypical lymphoid cells with hyperchromatic nuclei are seen in many areas of the interstitium (A). (Magnification, ×200) Immunohistochemical staining shows the lymphoid cells to be positive for B-cell markers CD20 (B) and large B-cell lymphoma markers mum-1 (C). (Magnification, ×100) Immunohistochemical staining using anti-CD34 shows the accumulation of lymphoid cells to be localized within the peritubular capillary lumina (D). (Magnification, ×400)

The day after admission, a percutaneous renal biopsy was performed. Eleven glomeruli were present with no evidence of mesangial cell proliferation or capillary wall thickening. Island-like accumulations of medium to large lymphoid cells were observed in many areas of the interstitium, with round vesicular nuclei containing distinct basophilic nucleoli (Figure 2A). Immunohistochemical analysis showed the atypical lymphoid cells to be positive for B-cell markers CD20 (Figure 2B) and negative for CK, CD3, CD45RO and CD10. They were positive for large B-cell lymphoma marker mum-1 (Figure 2C). Immunohistochemical staining using anti-CD34 confirmed the accumulation of lymphoid cells to be localized within the peritubular capillary lumina (Figure 2D). Immunofluorescence revealed negative staining of IgG, IgA, IgM, C3 and C1q with no glomerular immune deposits. Electron microscopy did not detect any electron dense deposits, but did show a cluster of atypical lymphocytes encircled by a peritubular capillary wall.

Pathologic examinations of percutaneous renal biopsies confirmed the diagnosis of intravascular large B-cell lymphoma of Stage IV_E_. The patient returned to the local hospital for symptomatic treatment after diagnosis. Ten days later she was readmitted into Nanfang hospital due to sudden bilateral lower limb fatigue and inability to walk. She was treated with chemotherapy consisting of CHOP (cyclophosphamide 1.0 g D1+Doxorubicin 60 mg D1+leurocristime 2 mg D1+Prednisone 100 mg D1-5) for a week. Palliative radiotherapy DT 40Gy/20F and other supportive treatment were provided for metastatic foci in the medullary cavity of sternum, T1-7. The patient regained muscle strength in both lower limbs and was able to walk again. She was discharged after hepatic and renal function values had returned to normal. Follow-up data shows the patient to be alive, nine months after discharge.

## Conclusions

Intravascular Large B-cell Lymphoma (IVLBCL), also described as intravascular lymphomatosis, angiotropic large cell lymphoma, and malignant angioendotheliomatosis, is a very rare subtype of Non-Hodgkin's lymphoma. The true incidence is unknown and there is no known sex predilection. The prognosis is usually very poor despite use of chemotherapy. Most patients present with fever of unknown origin and nonspecific cutaneous and neurologic manifestations. The renal involvement with IVLBCL has been classified as either intraglomerular growth or tubulointerstitial invasion. Microscopically, it is characterized by the proliferation and infiltration of lymphoma cells in capillaries and venules. No obvious extravascular tumor mass or detectable circulating tumor cells are observed in the peripheral blood.

The clinical manifestations include symptoms related to multi-organ failure [5]. In the past, the diagnosis was usually made at autopsy. With increased awareness and improved physical health check-ups more cases have been detected during life allowing for active and effective treatment.

The first case with IVLBCL was diagnosed by renal biopsy in 1981 [1]. Since then, several other studies have reported similar cases [6-8]. The common pathologic finding is localization of the lymphoma cells within the lumina of the glomerular capillaries. Bilateral marked nephromegaly is the other major clinical feature in patients with the tubulointerstitial type of disease. Renal size has been reported to improve rapidly in response to chemotherapy. Clinical findings associated with IVLBCL are reported to be acute renal failure (ARF) and nephritic-range proteinuria. The ARF might be caused by the obstruction of glomerular circulation due to the invasion of the lymphoid cells, as has been suggested in acute proliferative glomerulonephritis, resulting in a loss of renal function. The greater the proportion of glomeruli that is affected by the infiltration of tumor cells, the more the renal function has deteriorated, consistent with the view that the presence of tumor cells impair glomerular function. In the IVLBCL cases with nephrotic range proteinuria, histology of renal biopsies revealed minimal glomerular lesions because of the absence of immune deposits in the glomeruli.

In the present case, lymphoma cells involved multiple loci including the kidney, skeletal system, lungs, lymph nodes, spleen, and pelvic cavity. The mechanism of renal dysfunction could be due to the obstruction of peritubular capillaries with increased post glomerular vascular resistance, compression of tubules, or modulation of the tubuloglomerular feedback mechanism. The impairment of the patient's skeletal and pulmonary systems resulted in movement dysfunction and interstitial pneumonia. Lymph node inflammatory proliferation is considered with multiple bilateral neck and mediastinum lymph nodes enlargement. This indicates the occlusion of lymphoma cells in the vasculature, causing thrombosis and multiple system dysfunctions [9]. The enlarged lymph nodes may block drainage of fluid that lubricates the pelvic cavity, causing effusion accumulations and symptoms such as lower limb edema, pelvic effusion, and anorexia.

In the present case, the tumor cell accumulation is demonstrated to be confined within the peritubular capillary walls, by anti-CD34 staining. These lymphoid cells are positive for B-cell markers CD20, CD79a, and PAX-5. They are also positive for mum-1 and bcl-6, suggesting origin from the active B lymphocyte. The immunohistochemical analysis confirmed the diagnosis of IVLBCL. Although the cause for highly selective peritubular capillary localization of the lymphoma cell is unclear, it is possible the large size of the lymphoma cell results in entrapment of tumor cells within the glomerular rather than peritubular capillaries. The examination of the bone marrow was not helpful in the diagnosis of this case unlike other cases reported in literature [10].

In conclusion, when tubulointerstitial cell infiltration is found in a patient with bilateral marked nephromegaly and renal dysfunction without proteinuria, intra-peritubular capillary lymphoma should be considered as a possible diagnosis.

## Consent

Written informed consent was obtained from the patient for publication of this Case Report and any accompanying images. A copy of the written consent is available for review by the Editor-in-Chief of this journal.

## List of abbreviations

ARF: Acute renal failure was abbreviated; IVLBCL: Intravascular large B-cell lymphoma; PET-CT: Positron Emission Tomography-Computed Tomography

## Competing interests

The authors declare that they have no competing interests.

## Authors' contributions

XB and JG collected the data and drafted the manuscript. GJ carried out the gross examination and final diagnosis. XL and LW carried out the immunohistochemical study. GW and NJ participated in the interpretation of data. All authors have read and approved the final manuscript.
